# A Comprehensive Environmental and Molecular Strategy for the Evaluation of Fluroxypyr and Nature-Derived Compounds

**DOI:** 10.3390/ijms26178209

**Published:** 2025-08-24

**Authors:** Ion Valeriu Caraba, Luminita Crisan, Marioara Nicoleta Caraba

**Affiliations:** 1 Faculty of Bioengineering of Animal Resources, University of Life Sciences “King Mihai I” from Timisoara, Calea Aradului, 119, 300645 Timisoara, Romania; valeriucaraba@usvt.ro; 2 ANAPATMOL Research Center, “Victor Babes” University of Medicine and Pharmacy of Timisoara, E. Murgu, 2, 300041 Timisoara, Romania; nicoleta.caraba@umft.ro; 3 “Coriolan Dragulescu” Institute of Chemistry, Mihai Viteazu Blvd., 24, 300223 Timisoara, Romania; 4 Cellular and Molecular Biology Department, “Victor Babes” University of Medicine and Pharmacy of Timisoara, E. Murgu 2, 300041 Timisoara, Romania

**Keywords:** fluroxypyr, enzymes, bacteria, fungi, natural compounds

## Abstract

This study evaluated the effects of different doses of the herbicide fluroxypyr on soil microbial communities under controlled laboratory conditions. Specific enzymatic activities ((dehydrogenase (DA), urease (UA), catalase (CA), phosphatase (PA)) and quantitative variations in bacterial and fungal populations were measured regarding key physico-chemical soil parameters (temperature, pH, electrical conductivity, moisture, organic matter, ammonium, nitrate nitrogen, and available phosphate content). The effects of the herbicide on the targeted parameters were dose- and time-dependent. Fluroxypyr induced a clear decrease in DA, CA, and PA during the first 14 days after administration, while UA showed a decrease in the first 7 days, followed by a slight increase starting on day 14, closely related to the applied dose. Microbial populations decreased in direct relation to the fluroxypyr dose. Organic matter content exhibited a positive correlation with DA, UA, CA, as well as with microbial populations. In addition, three natural compounds structurally similar to fluroxypyr were identified via 3D virtual screening, demonstrating potential herbicidal activity. Fluroxypyr can alter soil metabolic activity and disrupt microbial communities, thereby affecting soil fertility. Used as a reference in 3D screening, fluroxypyr helped identify three natural compounds with potential herbicidal activity as safer alternatives to synthetic herbicides.

## 1. Introduction

Modern agriculture is not possible without the use of herbicides, however, their improper and excessive use can have negative effects on soil structure and cultivated crops, lead to water and soil pollution, and pose potential risks to human health [1,2,3,4]. The reliance on herbicides in agricultural crops has increased significantly in recent years (e.g., between 2011 and 2022, over half of the pesticides applied were classified as herbicide groups) [5], even though there are studies that highlight significant effects of herbicides on crop productivity [6]. The soil is recognized as a reservoir for a diverse range of enzyme classes including: oxidoreductases (dehydrogenase, catalase, polyphenol oxidase), hydrolases (urease, phosphatase, amylase, protease, cellulase, invertase), lyases, ligases, transferases, and isomerases [7].

Soil enzymatic activities play a crucial role in the biochemical processes underlying the biogeochemical cycle of elements, ensuring the transformation of substances and energy within the soil ecosystems, as well as contributing to the degradation of toxic and harmful substances [8]. Consequently, soil enzymes can serve as valuable indicators and tools for evaluating soil quality, health status, and productivity within agricultural ecosystems [9,10,11,12].

The determination of soil enzymatic activities allows for the evaluation of soil’s microbial status and facilitates the rapid and accurate detection of the impacts caused by natural or anthropogenic disturbances (such as agricultural practices applied in the field, including pesticide application) [12,13,14]. Enzymatic activities, as bioindicators, provide a rapid and efficient response to even minor environmental and soil-related changes, compared to other indicators such as soil respiration or soil microbial community diversity, which also contribute to the assessment of soil quality, health, and fertility [15,16,17]. Soil enzymatic activities are significantly influenced by the applied pesticides (type of pesticide, structure of the biologically active compound, administered dose, timing of application, and exposure duration), as well as by the physicochemical characteristics and parameters of the soil. These variables can quantitatively and qualitatively affect the soil microbial community and the activities of micro-organisms in their interaction with pesticides. Among the key physicochemical parameters that define a specific soil type and influence the interaction between micro-organisms and herbicides are temperature, pH, conductivity, soil type, humus content, and the levels of nitrites, nitrates, ammonium, sulfur, phosphorus, etc. [18,19,20,21].

The application of herbicides in agricultural crops affects the organisms present in the soil, with more pronounced effects on microbial populations, and some studies have reported a negative impact with reductions exceeding 95% [22]. For the majority of herbicides used in agricultural crops, studies show a considerable reduction in microbial populations (bacteria, fungi, and actinomycetes), with certain cases reporting the complete elimination of more sensitive species [15,23,24,25]. The immediate effect resulting from the reduction of the microbial population is the alteration of enzymatic activities and biochemical processes occurring in the soil, caused by biotic and/or abiotic stress (herbicide application) [14,21,26]. Studies examining the effect of herbicide application on soil show contradictory results regarding the interaction of herbicides with soil enzymes or microbial populations, but most indicate harmful and inhibitory effects on these soil quality parameters [27,28,29,30,31,32]. Herbicides are an essential component in ensuring agricultural yields by effectively controlling weeds that compete with crop plants for resources [33]. The rise in global demand for food has led to their increasing use in conventional and industrial agriculture. Nevertheless, the extensive and often excessive application of chemical herbicides has caused substantial environmental damage and contributed to the development of herbicide-resistant weed populations [34]. This scenario poses significant risks to ecosystems and human health [35]. Consequently, recent years have witnessed intensified research efforts aimed at discovering alternative weed management strategies [36]. Although the chemical structure of herbicides is often similar to that of drugs, progress in identifying new mechanisms of action or new chemical classes (scaffolds) in the herbicide field is significantly slower than in pharmaceutical sciences. One potential explanation for this disparity is the limited exploration of the potential available chemical diversity within the herbicide domain [37].

In recent decades, computational methods [38,39] have become indispensable in the rational design and optimization of agrochemical compounds and modern drug discovery and development. Among these approaches, molecular docking [40,41] provides structural insights into the interaction between candidate herbicidal molecules and specific plant enzymes, while simultaneously assessing the potential to avoid interactions with homologous targets in humans and beneficial non-target organisms, such as bees. In parallel, pharmacophore modeling techniques [42,43,44] allow the abstraction of essential steric and electronic features responsible for herbicidal activity, guiding the design of novel scaffolds with improved safety profiles. Moreover, quantitative structure-activity relationship (QSAR) methodologies [45,46] establish predictive models correlating molecular descriptors with biological activity and toxicity endpoints, facilitating virtual screening and early toxicity assessment. The integration of artificial intelligence and machine learning algorithms [47,48] has significantly improved the accuracy of predictive models, facilitating the concurrent optimization of herbicidal activity and safety for non-target species through the analysis of extensive chemical and toxicological datasets. These computational strategies provide a rational alternative to traditional approaches, accelerating the development of herbicides and pharmacological compounds with high efficacy, human safety, and reduced environmental impact [49,50].

The main purpose of the laboratory-based experiments was to investigate the effects of different doses of fluroxypyr on the soil microbial community. This was achieved through the assessment of specific enzymatic activities (dehydrogenase, urease, catalase, phosphatase) and the quantification of bacterial and fungal population dynamics. The results of these studies are closely linked to the physico-chemical parameters at the treated soil level, in this regard, the following parameters were determined: temperature, pH, electrical conductivity (EC), soil-water content, organic matter content of soil (OM), ammonium nitrogen content (NH_4_-N), nitrate nitrogen content (NH_4_-NO_3_), and available phosphate content. The foundation of this study was the critical role played by the soil microbial community in biogeochemical cycles and its subsequent influence on soil fertility, which in turn affects agricultural productivity. Additionally, fluroxypyr, a well-characterized synthetic herbicide, serves as a reference molecule for identifying structurally similar natural compounds from the ZINC15 Natural Products database [51]. Candidate compounds were evaluated for their potential herbicidal potential through a 3D similarity search [52,53], a method that facilitates the identification of molecules with comparable spatial conformations to fluroxypyr, thereby suggesting comparable bioactivity. Subsequently, to assess potential toxicological risks and possible endocrine-disrupting effects, molecular docking simulations were conducted using human transthyretin (TTR). TTR is a hormone transport protein involved in the distribution of thyroid hormones and vitamin A [54]. This integrated strategy allows the selection of effective herbicidal candidates with low binding affinity for TTR, thus reducing the likelihood of adverse health risks. The experimental validation of the selected natural compounds as safe herbicides will be conducted in future research.

## 2. Results

### 2.1. The Impact of Herbicides on Enzyme Activity and Microbial Populations

The mean values of the parameters measured at 7 days, 14 days, 21, 120 days, and 28 days of incubation are shown in Table 1. For variables subjected to analysis of variance (ANOVA), the observed values ranged as follows: (i) dehydrogenase activity (DA) ranged from 0.11 for the 0.5x treatment group at 28 days to 0.86 for the control at 0 days; (ii) urease activity (UA) ranged from 1.89 in the control at 7 days to 5.38 in the control at 28 days; (iii) catalase activity (CA) ranged from 0.81 in the 2x treatment group at 14 days and 7.28 in the control at 28 days; (iv) phosphatase activity (PA) ranged from 21.65 in the 2x treatment at 14 days and 126.04 in the control at 28 days; (v) bacteria-forming units ranged from 4.30 in the 0.5x treatment at 28 days to 10.90 in the same treatment at 14 days; and (vi) fungi-forming units ranged from 4.34 in the 0.5x treatment at 28 days to 10.83 in the control at 0 days. Measurements at day 0 were considered equivalent to the control group, as no herbicide exposure had occurred at that time point. The datasets for these variables met the assumptions of normality (*p* ≥ 0.067) and homogeneity of variances (*p* ≥ 0.054). Consequently, the prerequisites for conducting a two-way ANOVA were satisfied, with herbicide dose and exposure time serving as the two independent factors.

The results of the two-way ANOVA test evaluating the effects of herbicide dose, exposure time, and their interaction on various biological parameters are shown in Table 2. Also, Table 2 includes dehydrogenase activity, urease activity, catalase activity, phosphatase activity, bacterial abundance, and fungal abundance. Both fluroxypyr dose and exposure duration significantly influenced these metrics, as did their interactions. Exposure time exhibited the most pronounced effect on dehydrogenase, urease, and catalase activities among the tested factors, with herbicide dose and the interaction term contributing relatively little to overall variance. In contrast, dose was the dominant factor for phosphatase activity; it explained over half of its variance, the highest dose effect observed among all variables. In the case of bacteria, the temporal factor was also the primary contributor to the observed variability. The time-dose interaction accounted for nearly as much variance, exerting a stronger influence than any other variable examined. In the case of fungal abundance, time remained the most significant factor; the time-dose interaction made a larger contribution to explaining the overall variance compared to enzymatic activities.

**Figure 1:**
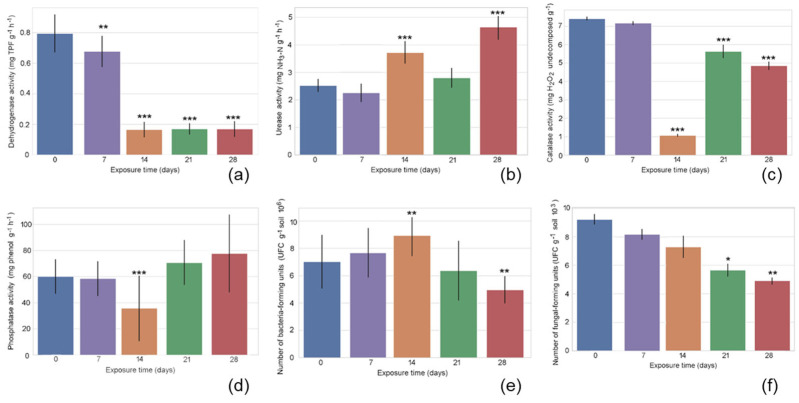
Temporal dynamics of dehydrogenase activity (**a**); urease activity (**b**); catalase activity (**c**); phosphatase activity (**d**); the number of bacteria colony-forming units (**e**); and the number of fungi colony-forming units (**f**). Data are expressed as mean values (box), with error bars indicating one standard deviation. Asterisks (*) indicate significant differences compared to the reference group (post-hoc Tukey HSD, ***—*p* ≤ 0.001, **—*p* ≤ 0.01, and *—*p* ≤ 0.05).

The marginal means for exposure duration are shown in Figure 1, illustrating the adjusted average response of the measured variable while controlling for the effects of other factors in the model. Dehydrogenase activity showed a significant decline at 7 days and 14 days, followed by stabilization at 21 days. Urease activity remained low and stable at days 0 and 7, showed a significant increase at 14 days, remained relatively constant at 21 days, and reached its peak at 28 days. Catalase activity revealed a significant six-fold decrease at 14 days, followed by a partial recovery at 21 days and again a decline at 28 days—both time points still showing activity levels below initial values. Phosphatase activity decreased significantly at 14 days, followed by an increase that exceeded baseline levels. Bacterial counts increased significantly by 14 days, followed by a sharp decline to significantly reduced levels at 28 days. Among all the variables examined, fungi abundance displayed the most distinct temporal trend: it progressively declined over time, with significantly lower levels observed at 21 days and 28 days.

**Figure 2:**
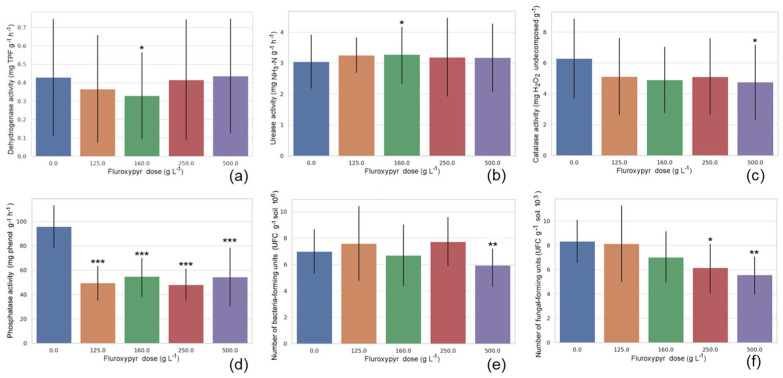
Effect of fluroxypyr dose on: dehydrogenase activity (**a**); urease activity (**b**); catalase activity (**c**); phosphatase activity (**d**); the number of bacteria colony-forming units (**e**); and the number of fungi colony-forming units (**f**). Data are expressed as mean values (box), with error bars indicating one standard deviation. Asterisks (*) indicate significant differences compared to the reference group (post-hoc Tukey HSD, ***—*p* ≤ 0.001, **—*p* ≤ 0.01, and *—*p* ≤ 0.05).

The marginal means for herbicide dose are shown in Figure 2. Dehydrogenase activity showed a U shape, with a significant decrease observed at the lowest effective dose (160 g L^−1^). Urease activity fluctuated within a narrow range but showed a statistically significant, albeit modest, increase at the same dose. Catalase activity tended to decrease with fluroxypyr dose, with this difference reaching statistical significance for the double maximum effective dose (500 g L^−1^). Phosphatase activity exhibited a clear and consistent dose-dependent decline in response to fluroxypyr application; the measured values showed a significant, up to ~40% decrease across all doses. While the number of bacterial colony-forming units remained relatively stable across low and intermediate fluroxypyr doses (0–250 g L^−1^), a significant decrease occurred at the highest dose (500 g L^−1^). The number of fungal colony-forming units is low with an increasing dose, showing significantly lower counts at both the maximum effective dose (250 g L^−1^) and the highest treatment level (500 g L^−1^).

To ensure that the interpretation of interaction, effects reflected both statistical and practical relevance, only interactions contributing more than 15% to the explained variance in the response variable were considered. This threshold was chosen to emphasize effects with substantial explanatory power and potential biological or ecological importance. Bacterial abundance and fungal abundance were the only variables meeting this criterion. A significant increase in bacterial counts was observed at 14 days for the lowest dose (125 g L^−1^), followed by a general decline by day 28, significantly reduced numbers of bacteria colony-forming units were detected for half maximal effective concentration (125 g L^−1^) and the highest dose (500 g L^−1^) found at 21 days and 28 days. Fungal abundance showed a decreasing trend between day 7 and day 14 with significant declines at the three highest doses (160–500 g L^−1^), By day 7 and 14, a decreasing trend was observed, with the most pronounced declines at 160–500 g L^−1^ (e.g., 4.37 CFU at 250 and 500 g L^−1^ on day 14, *p* ≤ 0.01). Bacterial colony-forming unit counts remained consistently lower in treated groups during later stages (21 and 28 days), although these differences were generally not statistically significant.

**Figure 3:**
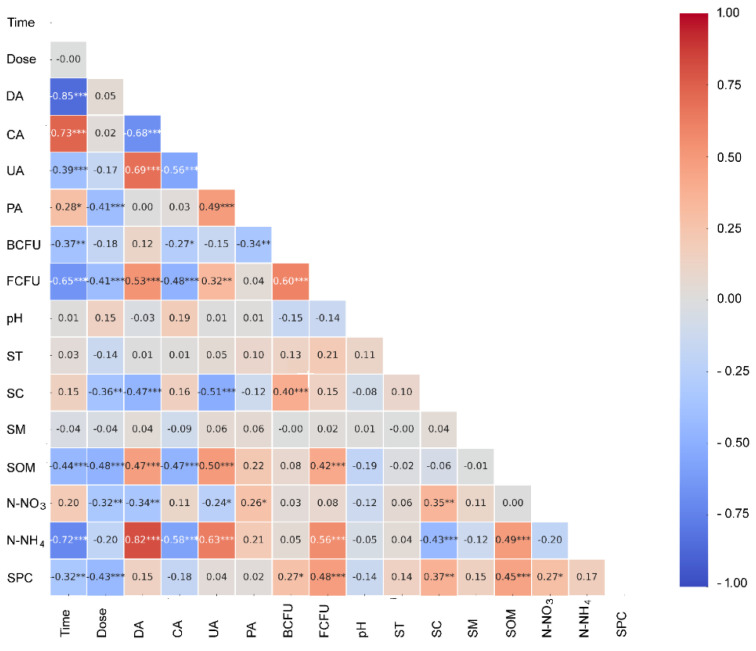
Heatmap of Pearson’s correlation coefficients between the analyzed variables. DA, dehydrogenase activity; UA, urease activity; CA, catalase activity; PA, phosphatase activity; BCFU, number of bacteria-forming units; FCFU, number of fungal-forming units; ST, soil temperature; SC, soil electrical conductivity; SM, soil moisture; SOM, soil organic matter content; N-NO_3_, nitrate nitrogen; N-NH_4_, ammonium nitrogen; SPC, soil phosphate content. Values are given as means with one standard deviation (in parentheses). Pearson correlations, ***—*p* ≤ 0.001, **—*p* ≤ 0.01, and *—*p* ≤ 0.05.

Pearson correlation heat maps are presented in Figure 3, showing the relationships among the analyzed variables. Exposure duration exhibited a very strong negative correlation with urease activity. Relationships of similar direction but lower magnitude were found for catalase activity, the number of fungal colony-forming units, organic matter content, and ammonium nitrogen concentration. This indicates a linear decline in these parameters over the experimental period. In contrast, urease activity correlated directly with time. Phosphatase activity showed a similar trend in relation to exposure duration, although this association was weaker.

Herbicide dose revealed weak inverse correlations with phosphatase activity, number of fungal colony-forming units, electrical conductivity, organic matter content, and phosphate content. These correlations indicate that higher doses are generally associated with lower values of these parameters. Additionally, significant associations were identified among enzymatic parameters. Specifically, dehydrogenase activity correlated inversely with urease activity and directly with catalase activity. Catalase activity was negatively associated with urease activity, while showing a positive correlation with phosphatase activity. The most pronounced correlations were found involving dehydrogenase activity.

Dehydrogenase activity showed a positive correlation with the number of fungal colony-forming units, soil organic matter content, and ammonium nitrogen concentration. However, it displayed inverse associations with soil electrical conductivity and nitrogen nitrate level. Urease activity was positively correlated with organic matter content and ammonium nitrogen concentration, a reverse pattern relative to dehydrogenase activity. Soil catalase showed strong positive associations with both soil organic matter content and ammonium nitrogen concentration, but a reverse correlation with soil electrical conductivity. Phosphatase activity revealed only weak correlations: a negative correlation with the number of bacteria colony-forming unit counts and a positive correlation with nitrate nitrogen.

Bacteria and fungi colony-forming units exhibited a strong positive correlation. Bacteria also showed similar but weaker relationships with soil electrical conductivity and phosphate content. Fungal abundance demonstrated direct correlations with soil organic content, ammonium nitrogen, and phosphate content. Organic matter content was strongly and positively correlated with the latter two metrics, emphasizing its role in nutrient retention and availability. Soil conductivity disclosed significant positive correlations with both nitrate nitrogen and soil phosphate content, while exhibiting a highly significant negative correlation with ammonium nitrate. These correlations suggest complex ionic dynamics in response to treatments. In contrast, the soil pH, temperature, and moisture showed weak and non-significant linear correlations with most other variables, indicating their relative stability in linear association with the dynamic changes observed in other parameters.

### 2.2. ADMETox Parameters for Fluroxypyr

In silico, prediction for ADMETox parameters is crucial as it provides preliminary insights into the pharmacokinetic properties and toxicity profiles of chemical compounds [55]. Detailed information regarding the significance of all calculated physicochemical, medicinal chemistry, and ADMETox parameters (Supplementary Materials Table S1) using ADMETlab3 is available at the following web address: https://admetlab3.scbdd.com/explanation/#/a (accessed on 24 September 2024).

The alerts structure for fluroxypyr outlines specific substructures that may be associated with potential toxicity or safety concerns. This assessment is based on Toxicophore Rules and Medicinal Chemistry principles, as presented in Table 3.

### 2.3. Three-Dimensional-Shape Similarity

**Figure 4:**
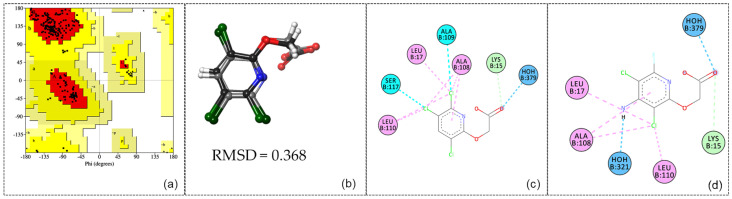
Analysis of the molecular docking procedure: Ramachandran plot (**a**); overlay of triclopyr (displayed in light gray) and fluroxypyr (rendered in dark gray) molecules (**b**); 2D molecular docking representation of triclopyr (**c**); and 2D molecular docking representation of fluroxypyr (**d**).

The provided coefficients obtained from ROCS analysis demonstrate a significant overall similarity between the triclopyr and fluroxypyr molecules, mainly in terms of shape and specific structural features (Figure 4b, Table 4).

The TanimotoCombo value (1.409) and FitTverskyCombo (1.511) suggest a significant degree of similarity, while the ShapeTanimoto score (0.886) further confirms this alignment in geometric conformation. The elevated RefTverskyCombo (1.769) and RefTversky (0.981) values emphasize that the molecules share important reference characteristics. However, the ColorTanimoto score (0.523) and the ColorScore (−4.808) suggest notable differences in their chemical attributes (e.g., the presence of an amino group at the 4-position and a fluorine atom at the 6-position on the pyridine ring of fluroxypyr compared to triclopyr). Despite these discrepancies, the high overlap value (583.116) indicates that the molecules occupy a considerable shared spatial region, supporting their potential for similar biological interactions. This hypothesis will be further investigated in the subsequent step through a molecular docking study to elucidate the specific binding modes of fluroxypyr with human transthyretin (TTR).

### 2.4. Molecular Docking Analysis

The UCLA-DOE LAB—SAVES v6.1 server was utilized to validate the human transthyretin structure (PDB ID: 5L4M) prior to conducting molecular docking studies. The ERRAT analysis yielded an excellent Overall Quality Factor of 93.4579, indicating high structural reliability. Furthermore, the Ramachandran plot (Figure 4a), assessed with PROCHECK, showed 92.0% of residues in the core region, 8.0% in the allowed region, and no residues in the generously allowed or disallowed regions. These results reflect the high quality of the 5L4M structure and confirm its suitability for use in the subsequent molecular docking investigation.

The 5L4M receptor was prepared as a dimer for molecular docking using the Protein Preparation Wizard and Receptor Grid Generation tools from the Schrödinger suite. Subsequently, triclopyr was docked, and the RMSD was computed between the reference triclopyr (RX) and the docked triclopyr to validate the docking procedure (Figure 4b). After successful validation, with an RMSD value of 0.368 and the reproduction of RX-triclopyr interactions with LYS15, LEU17, ALA108, ALA109, LEU110, SER117, and HOH379 (Figure 4c), molecular docking of fluroxypyr was conducted using the GLIDE module in Standard Precision (SP) mode.

The analysis of the docking results for fluroxypyr (Figure 4d) revealed two hydrogen bonds with formed water molecules HOH 321 and HOH 379, one hydrogen bond with LYS15, and hydrophobic interactions involving LEU17, ALA108, and LEU110. When comparing the docking poses of fluroxypyr and triclopyr (Figure 4c,d), the interactions observed for both ligands are closely similar, which is consistent with their high structural similarity (TC = 1.409). In the comparative analysis of fluroxypyr and triclopyr, the docking scores (gScore) show values of −7.021 for fluroxypyr and −6.698 for triclopyr, indicating that fluroxypyr exhibits a stronger binding affinity, although the difference is quite close to that of triclopyr. The Glide ligand efficiencies were reported as −0.468 for fluroxypyr and −0.478 for triclopyr, indicating a slightly lower efficiency for fluroxypyr. Additionally, the Glide van der Waals energies (evdw) were −21.923 for fluroxypyr and −25.111 for triclopyr, reflecting more favorable van der Waals interactions for triclopyr. The total Glide energies were −33.812 for fluroxypyr compared to −33.508 for triclopyr, further suggesting a greater binding stability for fluroxypyr. Overall, these findings indicate that both compounds exhibit strong binding characteristics, with fluroxypyr showing a marginally higher binding affinity, though the values are closely comparable.

### 2.5. Identification of Natural Product Analogs of Fluroxypyr via Virtual Screening

Virtual screening, as an efficient computational approach, was employed to rapidly identify potential natural compounds from the ZINC database with structural and chemical similarity to fluroxypyr. Natural compounds were chosen due to their structural diversity and proven biological activity, offering a valuable source for discovering novel and potentially safer herbicide analogs.

Compounds exhibiting relevant similarities in shape and chemical properties were selected to identify new candidates with similar biological activity (Supplementary Materials Table S2). This strategy supports the prioritization of molecules for subsequent experimental investigations, thereby improving the efficiency of time and resource utilization.

**Figure 5:**
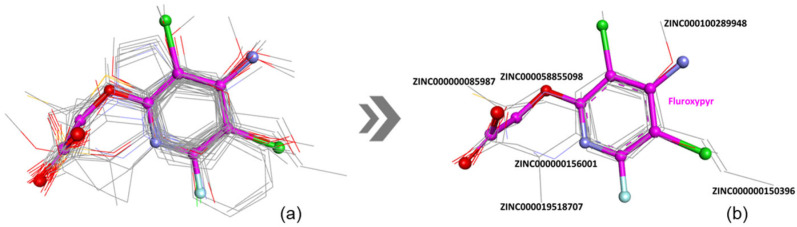
Overlay of the top compounds based on 3D similarity (TC score greater than 1.3) (**a**); Overlay of the six best compounds according to docking scores (**b**). Fluroxypyr is shown in magenta.

During the virtual screening aimed at identifying structural and chemical analogs of fluroxypyr, the TanimotoCombo (TC) score was used as the primary selection criterion. Molecules exhibiting a TC score greater than 1.3 (Table S2, Figure 5a) were prioritized, as this threshold reflects a substantial degree of similarity in both molecular shape and chemical properties relative to the fluroxypyr molecule.

The top-ranked compounds were subsequently subjected to molecular docking using Glide to evaluate their binding affinity toward human TTR. Under the assumption that weaker binding affinity corresponds with a reduced risk of interfering with TTR function, twenty-five compounds exhibiting lower binding affinities (gScore values ranging from −6.994 to −4.787) compared to fluroxypyr were selected for further analysis. Among these, six compounds were selected based on consistently inferior performance across multiple docking metrics, including the lowest glide gscore (gScore), reduced ligand efficiencies (LE), less favorable van der Waals interaction energies (Evdw), and higher total Glide energies (E). These results suggest a lower predicted binding affinity and less optimal interaction profiles with the TTR binding site, as summarized in Table 5.

Following a detailed analysis of Table 5, three compounds consistently demonstrated unfavorable docking parameters: ZINC000000156001 exhibited both the lowest ligand efficiency (LE = −0.173) and the weakest overall GlideScore (gScore = −4.787), indicating a notable unfavorable binding profile; ZINC000000085987 displayed the least favorable van der Waals interaction energy (Evdw = −18.017), suggesting minimal stabilizing interactions with the binding site, despite a moderate ligand efficiency (LE = −0.367); ZINC000058855098 presented a combination of a weak LE (−0.246) with an unfavorable Evdw (−20.169), reinforcing its classification as a poor binder.

**Figure 6:**
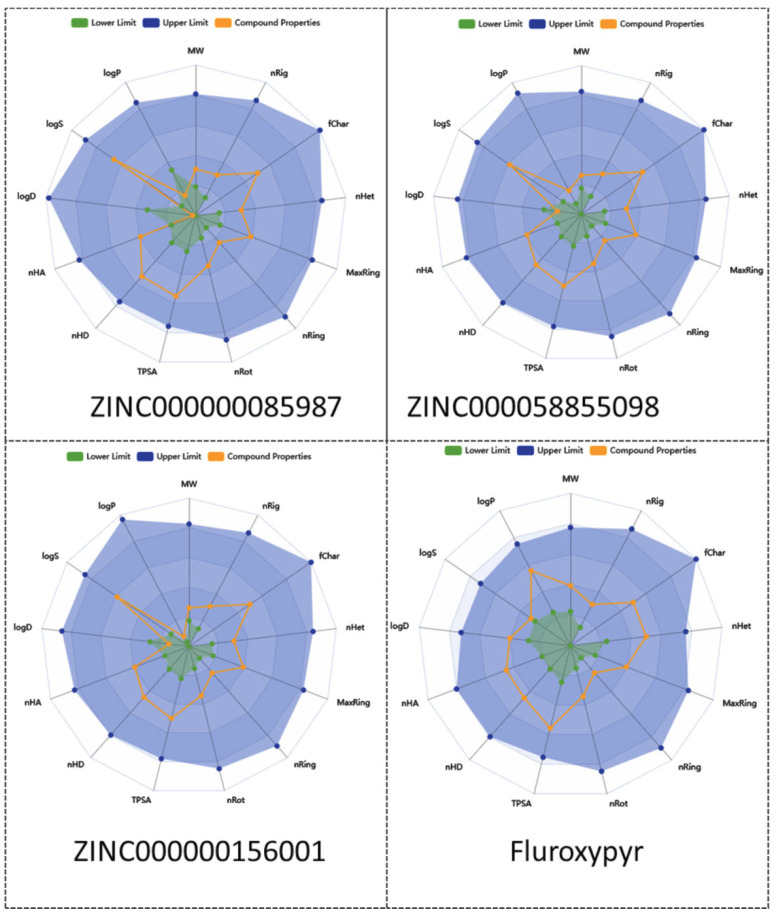
Radar plot of the analyzed compounds.

Further, a radar plot (Figure 6) was generated with ADMETlab3 based on key physicochemical properties, including lipophilicity (LogP), LogS (solubility), LogD (distribution coefficient), number of hydrogen bond acceptors (nhA), number of hydrogen bond donors (nHD), polar surface area (TPSA), rotatable bonds (nRot), ring counts (nRing, MaxRing), heteroatoms (nHet), fraction of sp3 carbons (FChar), and molecular weight (MW), revealing profiles that are comparable to those of fluroxypyr.

As their predicted interactions are energetically and structurally less favorable, these compounds exhibit a lower potential to interfere with TTR function and may serve as effective herbicides with comparable activity to fluroxypyr while presenting a lower risk to human safety. These candidates will be evaluated through experimental studies in future work to confirm their herbicidal efficacy and safety profiles.

## 3. Discussion

The application of herbicides to agricultural crops typically results in both quantitative and qualitative changes in the microbial community, alterations in soil metabolic activity, and disruptions in soil enzymatic functions. Among the most significantly affected enzymatic activities are dehydrogenase, urease, catalase, and phosphatase activities. The results concerning the effects of fluroxypyr on the soil microbiota and its associated activity, as observed in our study, are consistent with these previously reported findings. Soil enzymes are involved in biochemical processes that facilitate the breakdown and degradation of a wide variety of pollutants, including herbicides [3,16,56].

Dehydrogenase is an intracellular enzyme that plays a key role in energy production through the oxidation of organic compounds at the cellular level. Dehydrogenase activity is widely recognized as the most important indicator for assessing the metabolic activity of viable microbial communities in the soil. It is considered a marker enzyme for assessing and revealing the impact of pollutants on soil microbiota [57,58,59]. The inhibitory effects of herbicides on dehydrogenase activity have been extensively documented in the literature, with studies demonstrating variations in the degree of inhibition depending on the type of herbicide, the applied dose, and the incubation period [3,4,59,60].

The results of our study indicate that in soil samples treated with fluroxypyr, dehydrogenase activity decreased during the first 21 days following application and remained relatively stable thereafter until the end of the experiment period. The observed decreases in DA were dose-dependent, becoming apparent at a concentration of 160 g L^−1^. It is noteworthy that previous studies have reported a positive correlation between the decline in dehydrogenase activity and the reduction in soil microbial populations after herbicide application, affecting both bacterial and fungal communities [24,60,61]. The findings of this study support the aforementioned observations but contradict other studies reporting increases in dehydrogenase activity. This discrepancy is explained by the ability of soil fungi to produce dehydrogenase, which may contribute to the degradation of the herbicide [4,62,63].

The results of the present study align with previously published findings, demonstrating reductions in dehydrogenase activity (DA) that depend on both the incubation period and, more importantly, on the administered fluroxypyr dose. DA levels were found to correlate with bacterial and fungal populations, as well as with soil organic matter and ammonia content. Previous studies report the presence of micro-organisms capable of surviving and thriving in soils treated with herbicides; these micro-organisms exhibit resistance or participate in using herbicides as sources of carbon and energy. Nevertheless, sustained decreases in DA can impair the ability of microbial communities to degrade herbicides present in the soil, thereby prolonging their toxic effects [64]. Catalase is an enzyme from the oxidoreductase group involved in the decomposition of hydrogen peroxide [65] and is considered a biomarker for assessing microbial activities, especially those of aerobic micro-organisms [66]. Research examining the effects of herbicide application on catalase activity reports significant decreases, often irreversible over time, depending on the type of herbicide and the administered dose [61,66,67].

The observed decline in enzymatic activity is considered to result from the reduced abundance of the microbial community, particularly among herbicide-sensitive micro-organisms. The findings of this study are consistent with previously published reports, with DA showing significant decreases from the early stages post-application, persisting for up to 28 days after application. Enzymatic activity tends to decrease progressively with increasing fluroxypyr.

Urease is an intra- or extracellular enzyme involved in the hydrolysis of urea and, consequently, in the nitrogen cycle within the soil [16]. Studies on urease activity (UA) in soils treated with herbicides indicate that values remain relatively constant until the end of the experiment [4,68] or show slight increases in UA levels [69,70] as the applied herbicide dose increases [71,72]. The findings of our study indicate slight increases in urease activity (UA) in soil samples treated with fluroxypyr, with these increases being associated with and correlated to the organic matter and ammonia content in the soil. Furthermore, the scientific literature reports varying effects of herbicide application on soil UA, ranging from no effect [73] to mild inhibition [74] and even to strong inhibition in certain cases [72,75].

Alkaline phosphatase is a phosphomonoesterase belonging to the broad group of phosphatase enzymes involved in the phosphorus cycle and is present in the soil both intracellularly and extracellularly [16]. Data from the scientific literature report varying results regarding the interaction between herbicides and phosphatase activity. Both stimulatory effects on phosphatase activity have been observed [24,68,76] and have inhibitory effects specifically on alkaline phosphatase activity [56,77].

The results of our study indicate reductions of up to 40% in alkaline phosphatase (PA) activity across all applied fluroxypyr doses, which is consistent with previously reported data. Moreover, a weak negative correlation was observed between PA activity and the bacterial population in the analyzed soil, results that are in agreement with those reported in other studies [78].

Quantitative variations were observed in the bacterial and fungal populations of soil treated, indicating that both incubation time and herbicide dose contribute to these changes. The abundance of bacteria and fungi in soil samples decreased from day 14 until the end of the experiment. Moreover, increasing the administered fluroxypyr dose led to reductions in bacterial and fungal populations, which in turn caused decreases in enzymatic activities, with dehydrogenase (DA) and alkaline phosphatase (PA) being the most affected. These findings are consistent with those reported by other research groups, as the reduction of bacterial and fungal abundance in herbicide-treated soils has been extensively studied [79,80,81].

The observed reductions in microbial populations and enzymatic activities underscore the significant impact of fluroxypyr on soil biological health. Building on these findings, this study also implemented an integrated virtual screening approach that combined 3D shape similarity search (ROCS) with molecular docking to identify natural compounds with herbicidal potential comparable or superior to fluroxypyr, while also offering improved safety for human health. Screening of the ZINC15 database for compounds with Tanimoto Combo (TC) scores above 1.3 allowed us to focus on molecules structurally similar to the reference herbicide, thereby facilitating the identification of promising candidates.

Comparative analysis of fluroxypyr and the related herbicide triclopyr revealed structural differences, such as the presence of an amino group at the 4-position in fluroxypyr, which enhances solubility and biological interactions. Additionally, the fluorine atom at the 6-position may confer distinct electronic characteristics, influencing the compound’s reactivity and binding affinity. These structural variations may lead to differing mechanisms of action and efficacy when interacting with target proteins, underscoring the importance of comparing these two herbicides (fluroxypyr and triclopyr), which is critical in understanding their respective roles in biological systems. Through the analysis of fluroxypyr, we obtain a comprehensive understanding of its potential interactions and effects, particularly in relation to human transthyretin (TTR) binding. While this in silico analysis provides a valuable preliminary assessment, experimental tests are needed to confirm or contest these findings, ensuring a thorough assessment of the safety and efficacy of fluroxypyr in real biological systems.

Molecular docking against human TTR, a key hormone transport protein, provided insights into potential toxicological and endocrine-disrupting risks associated with these compounds. Notably, several natural compounds demonstrated weaker binding affinities to TTR compared to fluroxypyr, indicating a potentially lower risk for adverse effects, while in certain cases exhibiting better herbicidal properties. This early de-risking approach, supported by ADMET profiling, facilitated the prioritization of natural herbicide candidates that combine efficacy with improved safety profiles.

However, these in silico findings represent a preliminary assessment and require experimental validation to confirm the efficacy and safety of these compounds under actual biological conditions. Overall, integrating 3D-based similarity searching, molecular docking, and ADMET analysis provides a powerful and efficient workflow for identifying natural herbicide candidates with enhanced activity and reduced health and environmental risks, thereby contributing to the development of sustainable agricultural solutions.

## 4. Materials and Methods

### 4.1. Herbicides

**Figure 7:**
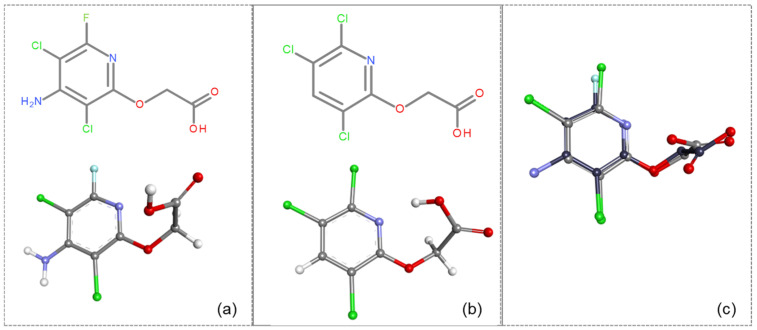
Two-dimensional (depicted in line) and three-dimensional (represented in ball-and-stick) structure of fluroxypyr (**a**); 2D and 3D structure of triclopyr (**b**); 3D-shape superposition of fluroxypyr (rendered in dark gray) and triclopyr (displayed in light gray) (**c**).

The experiments were conducted using the product available on the local market under the name Tomigan 250EC (Adama Agricultural Solutions Ltd., Glissando, Timisoara, Romania), a suspension containing fluroxypyr as the active substance at a concentration of 250 g L^−1^. Tomigan 250EC is a selective herbicide used to control annual and perennial broadleaf weeds in cereal and corn crops. Fluroxypyr is a selective, post-emergent, systemic herbicide, commonly known as 4-amino-3,5-dichloro-6-fluoro-2-pyridyloxyacetic acid, and is primarily registered for the control of broadleaf weed areas. Structurally, fluroxypyr belongs to the pyridine carboxylic acid class of herbicides and is characterized by having a fluorine atom at the 6-position on the pyridine ring (Figure 7a) distinguishing it from other herbicides like triclopyr (Figure 7b). Fluroxypyr’s mechanism of action involves mimicking the plant growth hormone auxin, thereby disrupting normal plant growth processes [82].

The fluroxypyr is generally considered safe under normal application conditions. However, human exposure may occur through inhalation, dermal contact, and ingesting contaminated food or water, raising potential health concerns, particularly regarding its weak binding to human transthyretin (TTR). This binding is relevant given that other herbicides in its class have been associated with thyroid-related neurotoxicity and developmental toxicity in animal studies. The detection of fluroxypyr in soil and groundwater across various countries underscores the need for careful monitoring and risk assessment to mitigate potential adverse effects on both human health and the environment.

### 4.2. Soil Sampling and Treatment

The soil samples used in the experiment were collected in May 2024 from a field located near the Ghiroda village (western Romania), at the following coordinates: latitude 45°53′13.14″ N, longitude 21°27′16.26″ E. The area is known to be free from pesticide or other xenobiotic applications. Chernozem samples were collected from the topsoil layer (0–20 cm) at six different points, then combined into a single 30 kg composite soil sample. This sample was homogenized by processing into fine particles and sieving through a 4 mm mesh. The processed soil was then divided into 5 kg portions for each of the five experimental variants.

The study included five experimental treatments: (i) T1-T4, representing soil samples treated with fluroxypyr; (ii) M, the untreated control sample. The selection of four different herbicide concentrations was based on the minimum and maximum application rates recommended by the manufacturer for agricultural use. Both reduced and double doses of fluroxypyr were applied to establish and analyze a comprehensive dose–response relationship and to assess potential environmental risks associated with herbicide overuse. The correspondence between the treatments and the manufacturer-recommended application rates is presented in Table 6.

The required herbicide doses were prepared by first creating a stock solution of the herbicide, followed by dilution with 500 cm^3^ of distilled water. The herbicide solutions were uniformly administered to the soil, distributed across the culture vessels, and thoroughly mixed to ensure homogenization and incorporation into the soil. The control soil sample was dispensed over the soil only with 500 cm^3^ of distilled water, without any herbicide added. Soil sampling was performed on days 1, 7, 14, 21, and 28 for each experimental variant, using a randomized sampling approach. After each sampling, the moisture content in the culture vessels was adjusted, and the soil was re-homogenized. The experiment was conducted under laboratory conditions for a period of 28 days, with temperature values ranging between 23.10 and 23.70 °C.

### 4.3. Physicochemical Properties of the Soil

The following physicochemical parameters of soil were monitored throughout the study: (a) temperature, pH, electrical conductivity (EC) were measured by using a handheld multimeter Multi 340i/SET WTW (Weilheim, Germany), fitted with a specific sensor for each parameter; (b) soil-water content; thermogravimetric analysis of samples was performed using the thermo-gravimetric method [83]; (c) water content, organic matter content of soil (OM) were determined by the calcination method [84]; (d) ammonium nitrogen content (NH_4_-N), nitrate nitrogen content (NH_4_-NO_3_), and available phosphate content were performed by measuring the absorbance using a spectrophotometer T90 UV/Vis (PG Instruments, Lutterworth, UK) at a wavelength of 630 nm, of 543 nm [85], 882 nm [86], respectively. A detailed description of the methodologies employed for the physicochemical parameters is presented in detail in other studies carried out by the research team [3,87].

### 4.4. Enzymatic Activity Analysis

The enzymatic activity, including dehydrogenase (DA), urease (UA), and alkaline phosphatase (PA) was determined with a T90 UV/Vis spectrophotometer (PG Instruments, Lutterworth, UK), Catalase activity (CA) was determined by the potassium permanganate (KMnO_4_) titration method.

Dehydrogenase enzymatic activity (EC 1.1.1.1) was evaluated using the organic salt 2,3,5-triphenyl tetrazolium chloride (TTC), dissolved in water as a clear solution, serving as the enzymatic substrate and final electron acceptor. Under the catalytic action of dehydrogenase, the substrate is reduced to triphenyl formazan (TPF), a red-colored compound whose absorbance is measured at 485 nm. Dehydrogenase activity is expressed as milligrams of TPF per gram of soil after 48 h of incubation [3,88].

Urease enzymatic activity (EC 3.5.1.5) was evaluated using a straightforward method based on the decomposition of urea into ammonia (NH_3_) and carbon dioxide (CO_2_). Enzymatic activity is expressed as mg NH_3_-N per gram of soil per hour (mg NH_3_-N g^−1^ h^−1^) after 24 h. The absorbance of the yellow-colored reaction product was measured at 445 nm [21,87,89].

Catalase enzyme activity (EC 1.11.1.6) was determined based on the measurement of O_2_ consumption following the addition of potassium permanganate (KMnO_4_) to a hydrogen peroxide (H_2_O_2_) solution. The remaining H_2_O_2_ in the supernatant solution was titrated with 0.05 M KMnO_4_, with the titration endpoint indicated by the formation of a pale pink solution. Catalase activity is expressed as mg of undecomposed H_2_O_2_ per gram of soil [90].

The evaluation of alkaline phosphatase enzymatic activity (EC 3.1.3.1) was based on the decomposition of phenylphosphate by the enzyme into disodium phenylphosphate and phenol. In the presence of 2,6-dibromoquinone-chloramide, these compounds react to form a blue-colored compound, which can be measured spectrophotometrically at 597 nm. Alkaline phosphatase activity was expressed as mg phenol per gram of soil after 48 h [3].

Additional details regarding the application of these experimental protocols were presented in our previous studies on the evaluation of the effects of other herbicides on soil enzyme activity [3,87]. For each experimental treatment from which soil samples were collected, all measurements were conducted in triplicate by the same researcher on the same day.

### 4.5. Microbiological Analysis

In order to evaluate the total number of bacteria and fungi colony-forming units (CFU)/g of soil, serial dilutions ranging from 10^−1^ to 10^−6^ were prepared starting from 1 g of soil obtained from herbicide-treated samples incubated under both laboratory and field conditions. Equal volumes of 100 μL of diluted soil suspension of 10^−6^ for bacteria and 10^−3^ for fungi were inoculated on selective nutrient culture medium: for bacteria, the culture medium was Plate-Count-Agars (Carl RothGmbH, Karlsruhe, Germany), and incubation was carried out at 37 °C for 48 h. The culture medium used for growing fungi was Potato-Glucose-Agar (PGA) (Carl RothGmbH, Karlsruhe, Germany), incubation was carried out at 28 °C for 72 h [3,91].

### 4.6. In Silico Evaluation of Fluroxypyr

The present in silico evaluation aimed to establish a comprehensive workflow protocol aimed at identifying compounds, specifically herbicides, that may interact with human transthyretin (TTR). Employing advanced computational methods (e.g., physicochemical and pharmacokinetic prediction and analysis, 3D-shape similarity search, and molecular docking) facilitates the systematic analysis of potential binding interactions between various herbicides and TTR. This approach provides valuable insights into the safety and potential risks associated with herbicide (fluroxypyr) exposure, allowing for more efficient identification of compounds that could disturb TTR function.

#### 4.6.1. ADMETox Characterization of Fluroxypyr

For the ADMETox (Absorption, Distribution, Metabolism, Excretion, and Toxicity) characterization of fluroxypyr, the ADMETlab 3.0 online-platform (https://admetlab3.scbdd.com/ accessed on 24 September 2024) [92], trained by the Directed Message Passing Neural Network (DMPNN) framework, was used to predict a series of pharmacokinetic and toxicological properties. The evaluation comprised the following categories: (i) Physicochemical & Medicinal Chemistry Properties: e.g., Predictions for molecular weight, LogP, solubility, and drug-likeness; (ii) ADME: e.g., Human intestinal absorption (HIA), Caco-2 permeability, plasma protein binding (PPB), CYP enzyme inhibition/substrate predictions; (iii) Toxicity Profile: e.g., Assessment of hepatotoxicity, hERG inhibition (cardiotoxicity), genotoxicity, carcinogenicity, and developmental toxicity across 36 toxicity endpoints; (iv) Toxicophore Screening: e.g., Identified potential toxic substructures based on eight toxicophore rules (751 substructures). This analysis provides an exhaustive evaluation of fluroxypyr’s potential health risks and environmental impact, ensuring a better understanding of its ADMET profile.

#### 4.6.2. 3D-Shape Similarity of Fluroxypyr

The 3D shape similarity between two molecules can assess how closely they resemble each other in three-dimensional space and is a crucial aspect of molecular modeling. Techniques such as ROCS (Rapid Overlay of Chemical Structures) [52] enable the alignment of molecular shapes, allowing for the evaluation of structural similarities based on 13 similarity coefficients (TanimotoCombo, ShapeTanimoto, ColorTanimoto, FitTverskyCombo, FitTversky, FitColorTversky, RefTverskyCombo, RefTversky, RefColorTversky, ScaledColor, ComboScore, ColorScore, Overlap). High shape similarity suggests that the molecules may bind similarly to a target protein, leading to comparable pharmacological effects. For ROCS analysis, the triclopyr extracted from the human transthyretin complex was used as a query molecule, while the fluroxypyr’s conformers were generated using the Omega v.2.5.1.4 module from the OpenEye suite www.eyesopen.com [93]. The default settings were maintained throughout the investigation. The same approach was applied using fluroxypyr as the query and omega-generated conformers from the ZINC database as the screening library. Investigating shape similarity serves as a valuable approach in ligand-based compound discovery and plays a crucial role in predicting the behavior of novel compounds based on known reference substances [94].

#### 4.6.3. Molecular Docking

In this study, human transthyretin (TTR) was selected as the target protein for docking fluroxypyr due to its role as a thyroid hormone transporter in vertebrates and its known interaction with thyroid hormone-disrupting chemicals [95]. Thyroid disruption caused by xenobiotics is associated with a broad spectrum of severe adverse outcomes, and TTR is a crucial molecular target for assessing such effects [96].

The crystal structure of human transhertin in complex with triclopyr (PDB ID: 5L4M, Resolution: 1.58 Å, R-Value Free: 0.198, R-Value Work: 0.160, R-Value Observed: 0.162 [96] was obtained from the Protein Data Bank (http://www.rcsb.org, accessed on 24 August 2024). This protein was chosen due to the high 3D similarity between the co-crystallized ligand (triclopyr, PDB ID: SBK) and fluroxypyr (see Section 4.6.2). Before protein preparation for docking, the protein structure was evaluated using the UCLA-DOE LAB-SAVES v6.1 web server (https://saves.mbi.ucla.edu/, accessed on 25 September 2024) to confirm its accuracy and reliability. This examination involved tools such as ERRAT [97] and PROCHECK [98]. Subsequently, the protein was prepared, refined, and analyzed using Schrödinger’s Protein Preparation module [99] with default options.

For the molecular docking simulation, the Schrödinger’s Glide module [100,101] was engaged. The grid box for 5L4M was generated using the Schrödinger’s Receptor Grid Generation tool by selecting the crystallized ligand, triclopyr, at the receptor’s active binding site. To validate the docking simulation, the X-ray ligand (triclopyr, PDB ID: SBK) from the human transthyretin receptor (PDB ID: 5L4M) was re-docked into the host protein, and the root-mean-square deviation (RMSD) value between the docked and extracted structures [102] was calculated to assess the precision of the docking protocol.

#### 4.6.4. Statistical Analysis

Data sets for dehydrogenase activity, urease activity, catalase activity, phosphatase activity, bacterial abundance (i.e., the number of bacteria-forming units), and fungal abundance (the number of fungi-forming units) for each combination of the groups of the two independent variables (fluroxypyr dose, exposure time) were tested for normality and homogeneity of variance. Kolmogorov–Smirnov tests and Bartlett’s tests were used for this purpose. For variables meeting these conditions, two-way ANOVAs were run, with dose and time being used as factors. For significant main effects, post hoc analysis was conducted using the Tukey HSD approach. These comparisons were performed against controls (dose as main effect) and the earliest time point (time as main effect). In cases of significant interactions between fluroxypyr dose and exposure duration, dose comparisons were run at each time point using the respective control group as the reference. The coefficient of determination (r^2^) is more commonly used in regression analyses to describe variance explained, but it is not the standard for correlation tables. Regarding the two-way ANOVA, a global R^2^ is not typically reported because variance is partitioned among multiple factors and their interaction. Instead, the recommended practice is to report effect sizes such as η^2^ or partial η^2^ for each factor: η^2^ × 100 ≈ Contribution (%). These measures more accurately represent the proportion of variance explained by each independent variable in multifactorial designs. Pearson’s correlations (r) were calculated to assess the overall trends and associations between all continuous measurements. The strength of these associations was interpreted according to the following guidelines: 0.00 ≤ r ≤ 0.29, weak correlation; 0.30 ≤ r ≤ 0.49, moderate correlation; 0.50 ≤ r ≤ 0.69, strong correlation; and 0.70 ≤ r ≤ 0.99, very strong correlation. All statistical analyses were conducted using Statistica version 10 (StatSoft Inc., Tulsa, OK, USA), with statistical significance defined as a two-tailed *p* ≤ 0.05.

#### 4.6.5. Limitations of the In Silico Study

Although computational methods are efficient and rapid, they present important limitations that must be considered when interpreting the results. Molecular docking [103] assumes limited flexibility of the receptor (the TTR protein), which may lead to underestimation or overestimation of true binding affinities under dynamic biological conditions. Moreover, simulations are performed under idealized conditions, neglecting cellular environment effects such as the presence of other proteins, pH variations [104], or post-translational modifications [105]. In silico ADMET filtering relies on predictive statistical models, which, while useful for triage, cannot fully replace experimental testing and clinical studies. Some toxicological or pharmacokinetic properties may not be accurately predicted, requiring further experimental validation [106]. Consequently, conclusions based solely on computational data must be validated through rigorous experimental methods to ensure the relevance and practical applicability of the selected compounds.

##### Future Perspectives and Recommendations

To validate and extend the findings obtained in this study, it is essential to pursue a series of experimental tests that confirm the herbicidal potential and safety of the identified compounds. In vitro biochemical assays, such as determining binding affinity to the human TTR protein and to enzymatic targets in weeds, will yield concrete data on interaction mechanisms and biological activity. Furthermore, biological testing on plants and under field conditions is necessary to evaluate actual herbicidal efficacy, the spectrum of activity, and potential adverse effects.

In parallel, integrating these approaches with advanced computational techniques and molecular dynamics simulations can enhance the predictive power of toxicological and herbicidal models. This integration can facilitate the more efficient identification and prioritization of compounds with reduced risk and improved efficacy. Moreover, interdisciplinary collaboration among chemists, biologists, and toxicologists is vital for developing modern, safe, and sustainable herbicides. Continuous monitoring of the ecological impact of proposed compounds is also recommended through ecotoxicity studies and long-term assessments in real ecosystems to prevent unforeseen adverse effects.

##### Connection to Sustainability

The identification and development of herbicides based on natural compounds or their derivatives can make a significant contribution to more sustainable and environmentally friendly agriculture [107,108]. Natural products generally exhibit better biodegradability and more favorable toxicological profiles, reducing the risks of accumulation in soil and water as well as adverse effects on non-target organisms [34,109].

By applying virtual screening combined with toxicological analysis focused on interactions with essential proteins, it is possible to select herbicidal compounds that are not only effective but also safer for human health and biodiversity. This strategy helps reduce the use of traditional chemical herbicides, which can sometimes be persistent and harmful [110,111].

Therefore, computational studies provide a powerful tool to accelerate research toward alternative, eco-friendly crop protection solutions [112,113,114] aligned with the principles of sustainable agriculture and the One Health concept (https://www.who.int/health-topics/one-health#tab=tab_1 (accessed on 4 July 2025)), which integrates the health of the environment, animals, and humans.

## 5. Conclusions

Our study provides a comprehensive and in-depth assessment of the effects of the herbicide fluroxypyr on soil microbiota, revealing dose and exposure time-dependent inhibitory effects that may lead to the destabilization of soil structure and fertility. Under the experimental conditions investigated, key soil enzymatic activities exhibited variations depending on the applied dose and exposure duration. The effects of fluroxypyr on DA were transient and dependent on both dose and time, with the highest inhibition rate observed within the first 14 days after application. Similarly, CA showed significant decreases with increasing fluroxypyr doses, with the most pronounced effects also occurring within the first 14 days post-application. The most evident inhibitory effects of fluroxypyr were observed on PA, with reductions of up to 40% compared to the initial values recorded on day 0, particularly at higher doses. The results emphasize a more pronounced inhibitory effect of fluroxypyr on PA and UA compared to DA, mainly due to the higher concentrations used relative to the normal dose. Among the enzymatic activities analyzed, UA showed a slight increase with increasing fluroxypyr dose, although values tended to decrease in the first 7 days after application. The bacterial and fungal populations in soil treated with fluroxypyr recorded reductions in CFU values as the herbicide dose increased, indicating a direct impact on the soil microbiota. Based on statistical analyses, significant correlations were established between enzymatic activities, bacterial and fungal populations, and certain soil physicochemical parameters. The most influential soil parameters affecting enzymatic activities and microbial populations were organic matter content, ammonium and nitrate nitrogen content, and available phosphorus content. Most herbicides applied in agricultural systems cause imbalances in soil that, over time, may negatively affect its fertility. Based on our findings, we recommend the controlled application of herbicides strictly within the doses recommended by manufacturers. Therefore, the study continued with 3D virtual screening using fluroxypyr as a reference, which led to the selection of three natural compounds with potential herbicidal activity to be tested in future studies as safer alternatives.

## Figures and Tables

**Table 1:** Mean values for the measured parameters at different doses and time points.

Treatment	Time	Dose	DA	UA	CA	PA	BCFU	FCFU	pH
UTC	0	0	0.85 (0.08)	2.78 (0.09)	7.82 (0.17)	84.62 (2.65)	5.13 (0.76)	9.33 (0.55)	7.96 (0.36)
0.5x	0	125	0.80 (0.08)	2.71 (0.15)	7.71 (0.21)	55.22 (2.54) ***	8.40 (0.78)	10.83 (1.29)	7.72 (0.29)
MinD	0	160	0.62 (0.17)	2.54 (0.14)	7.78 (0.19)	57.91 (1.92) ***	6.87 (2.50)	9.50 (0.46)	8.02 (0.48)
MaxD	0	250	0.84 (0.08)	2.36 (0.10)	7.49 (0.15)	51.95 (1.72) ***	9.20 (0.40)	9.43 (0.47)	7.75 (0.38)
2x	0	500	0.86 (0.05)	2.23 (0.05)	7.26 (0.11)	50.27 (0.04) ***	5.50 (0.66)	7.00 (0.53)	7.88 (0.22)
UTC	7	0	0.74 (0.10)	1.89 (0.06) ***	7.60 (0.12)	83.25 (3.00)	7.67 (1.17)	7.67 (1.17)	7.19 (0.22)
0.5x	7	125	0.59 (0.10)	2.81 (0.16) *	7.46 (0.14)	52.15 (1.53) ***	9.47 (1.82)	10.03 (1.63)	7.47 (0.38)
MinD	7	160	0.57 (0.03)	2.22 (0.03)	6.54 (0.04) ***	57.19 (0.26) ***	8.53 (3.14)	8.23 (1.53)	7.61 (0.09)
MaxD	7	250	0.75 (0.04)	2.13 (0.18)	7.16 (0.09) *	49.71 (0.36) ***	7.20 (0.40)	7.20 (0.40)	7.59 (0.18)
2x	7	500	0.73 (0.07)	2.19 (0.14)	7.07 (0.19) **	49.90 (3.06) ***	7.50 (0.96)	7.73 (1.17)	7.48 (0.12)
UTC	14	0	0.24 (0.05)	3.60 (0.12)	1.50 (0.21)	84.36 (0.04)	7.80 (1.64)	10.30 (0.36)	7.39 (0.32)
0.5x	14	125	0.16 (0.03) *	3.36 (0.24)	1.16 (0.13) *	24.54 (4.04) ***	10.90 (0.36) *	10.57 (0.25)	7.47 (0.12)
MinD	14	160	0.14 (0.02) *	3.66 (0.26)	1.08 (0.09) **	24.72 (0.02) ***	9.67 (0.81)	6.83 (2.64) *	7.98 (0.34)
MaxD	14	250	0.15 (0.04) *	3.65 (0.19)	0.82 (0.02) ***	23.85 (2.88) ***	8.47 (1.25)	4.37 (0.50) **	7.65 (0.12)
2x	14	500	0.14 (0.03)	4.35 (0.40) *	0.81 (0.03) ***	21.65 (0.04) ***	7.83 (0.50)	4.37 (0.50) **	7.89 (0.36)
UTC	21	0	0.16 (0.03)	2.64 (0.17)	8.04 (0.97)	100.68 (6.98)	8.03 (2.23)	8.03 (2.23)	7.75 (0.41)
0.5x	21	125	0.17 (0.04)	3.18 (0.12) *	4.91 (0.87) ***	66.61 (2.65) ***	4.87 (0.81) *	4.97 (0.95)	7.64 (0.13)
MinD	21	160	0.15 (0.03)	3.18 (0.14) *	5.50 (0.22) **	72.99 (3.46) ***	5.47 (0.83)	5.70 (0.82)	7.75 (0.23)
MaxD	21	250	0.14 (0.03)	2.42 (0.20)	5.34 (0.03) **	57.35 (0.01) ***	9.13 (0.68)	5.20 (1.82)	7.68 (0.12)
2x	21	500	0.22 (0.02)	2.57 (0.17)	4.36 (0.20) ***	55.90 (2.63) ***	4.33 (0.42) *	4.40 (0.46)	7.74 (0.41)
UTC	28	0	0.15 (0.03)	4.32 (0.11)	6.46 (0.21)	126.04 (2.30)	6.33 (0.90)	6.33 (0.90)	7.85 (0.27)
0.5x	28	125	0.11 (0.01)	4.21 (0.21)	4.30 (0.13) ***	49.08 (1.15) ***	4.30 (1.13) *	4.34 (1.12) *	7.53 (0.33)
MinD	28	160	0.16 (0.01)	4.78 (0.24)	4.56 (0.34) ***	61.45 (4.08) ***	4.93 (0.65)	4.87 (0.55)	7.84 (0.00)
MaxD	28	250	0.20 (0.04)	5.38 (0.16) *	4.67 (0.29) ***	57.68 (1.73) ***	4.67 (0.29)	4.63 (0.35)	7.74 (0.06)
2x	28	500	0.23 (0.04)	4.53 (0.18)	4.27 (0.24) ***	93.98 (6.03) ***	4.47 (0.67) *	4.40 (0.56)	7.82 (0.02)
**Treatment**	**Time**	**Dose**	**ST**	**SC**	**SM**	**SOM**	**N-NO_3_**	**N-NH_4_**	**SPC**
UTC	0	0	22.60 (0.46)	274.41 (2.95)	10.11 (1.21)	23.42 (0.69)	2.87 (0.44)	22.97 (0.30)	60.77 (2.44)
0.5x	0	125	22.23 (0.45)	275.01 (4.08)	8.13 (0.70)	22.02 (0.88)	2.68 (0.05)	22.85 (0.31)	55.60 (0.70)
MinD	0	160	22.10 (0.60)	272.13 (0.00)	7.58 (1.21)	19.86 (1.84)	2.86 (0.08)	22.88 (0.25)	50.20 (1.72)
MaxD	0	250	22.20 (1.01)	263.54 (3.27)	9.13 (0.72)	19.75 (0.23)	2.89 (0.26)	22.59 (0.33)	47.58 (2.26)
2x	0	500	22.50 (0.30)	271.42 (2.08)	8.95 (1.06)	18.83 (1.58)	2.75 (0.12)	21.98 (0.41)	52.38 (2.25)
UTC	7	0	22.13 (0.64)	277.10 (1.44)	9.06 (0.69)	24.09 (1.44)	2.79 (0.14)	22.76 (0.36)	57.28 (3.73)
0.5x	7	125	22.03 (0.74)	275.49 (1.53)	8.25 (0.41)	20.37 (0.57)	2.83 (0.13)	22.52 (0.28)	54.09 (2.82)
MinD	7	160	21.80 (0.40)	270.98 (0.74)	8.64 (0.26)	18.27 (0.85)	2.68 (0.11)	22.48 (0.41)	47.86 (6.89)
MaxD	7	250	22.53 (0.21)	266.45 (1.17)	9.51 (1.22)	19.26 (2.46)	2.59 (0.38)	22.18 (0.43)	37.07 (1.66)
2x	7	500	21.63 (0.67)	272.97 (0.41)	9.05 (0.81)	18.29 (0.68)	2.45 (0.02)	21.78 (0.15)	49.71 (2.33)
UTC	14	0	22.20 (0.95)	375.34 (0.85)	8.44 (0.68)	18.98 (1.21)	3.76 (0.31)	21.87 (0.20)	57.28 (2.51)
0.5x	14	125	22.77 (0.51)	342.59 (1.55)	9.28 (1.07)	19.04 (0.50)	2.82 (0.11)	20.78 (0.12)	56.02 (2.40)
MinD	14	160	22.37 (0.68)	336.57 (1.50)	9.47 (2.30)	16.84 (1.03)	2.95 (0.16)	20.88 (0.07)	53.94 (1.80)
MaxD	14	250	21.67 (0.40)	329.90 (0.44)	8.86 (2.54)	17.74 (0.22)	2.89 (0.30)	20.75 (0.35)	49.23 (1.47)
2x	14	500	21.97 (0.21)	319.43 (1.13)	8.30 (0.96)	18.02 (2.00)	2.76 (0.34)	20.81 (0.12)	47.58 (1.90)
UTC	21	0	22.43 (1.17)	368.61 (1.00)	9.69 (0.25)	20.66 (1.48)	3.13 (0.04)	21.09 (0.34)	56.38 (1.81)
0.5x	21	125	21.73 (0.35)	256.30 (0.53)	9.26 (0.52)	19.61 (0.17)	3.45 (0.13)	21.18 (0.33)	52.79 (1.65)
MinD	21	160	22.33 (0.90)	297.48 (1.12)	9.10 (0.67)	19.50 (0.63)	2.98 (0.30)	20.98 (0.29)	50.29 (1.06)
MaxD	21	250	22.60 (0.42)	289.09 (0.68)	10.68 (1.93)	19.06 (0.42)	2.75 (0.45)	20.97 (0.08)	50.60 (1.62)
2x	21	500	22.20 (0.62)	289.06 (1.61)	8.09 (1.25)	18.08 (0.36)	2.87 (0.34)	21.06 (0.23)	44.89 (3.35)
UTC	28	0	22.50 (0.80)	297.82 (0.80)	8.40 (1.33)	18.13 (0.78)	2.95 (0.30)	21.19 (0.44)	42.63 (2.23)
0.5x	28	125	22.23 (0.60)	288.45 (0.57)	7.55 (1.76)	19.57 (0.90)	2.75 (0.26)	21.18 (0.21)	42.10 (0.92)
MinD	28	160	22.43 (0.91)	288.32 (1.01)	7.67 (1.56)	17.95 (0.50)	2.64 (0.26)	21.56 (0.46)	50.35 (1.47)
MaxD	28	250	22.20 (0.44)	289.47 (1.22)	9.97 (1.04)	17.44 (1.89)	2.96 (0.12)	20.29 (0.20)	51.88 (1.63)
2x	28	500	22.10 (0.70)	216.67 (1.58)	9.01 (0.58)	16.43 (2.42)	2.88 (0.24)	21.74 (0.13)	41.63 (1.23)

UTC, untreated controls; 0.5x, half maximal effective concentration; MinD, minimal effective dose; MaxD, maximal effective dose; 2x, double maximal effective dose; DA, dehydrogenase activity; UA, urease activity; CA, catalase activity; PA, phosphatase activity; BCFU, number of bacteria-forming units; FCFU, number of fungal-forming units; ST, soil temperature; SC, soil electrical conductivity; SM, soil moisture; SOM, soil organic matter content; N-NO_3_, nitrate nitrogen; N-NH_4_, ammonium nitrogen; SPC, soil phosphate content. Values are given as means with one standard deviation (in parentheses). post-hoc Tukey HSD, ***—*p* ≤ 0.001, **—*p* ≤ 0.01, and *—*p* ≤ 0.05.

**Table 2:** Mean values for the measured parameters at different doses and time points.

Variable	Factor	*p*-Value	Contribution (%)
DA	Time	<0.001 ***	92.85
DA	Dose	<0.001 ***	2.01
DA	Time × Dose	0.006 **	2.3
UA	Time	<0.001 ***	86.12
UA	Dose	0.007 **	0.73
UA	Time × Dose	<0.001 ***	10.86
CA	Time	<0.001 ***	89.88
CA	Dose	<0.001 ***	5.12
CA	Time × Dose	<0.001 ***	3.9
PA	Time	<0.001 ***	33.39
PA	Dose	<0.001 ***	52.47
PA	Time × Dose	<0.001 ***	13.25
BCFU	Time	<0.001 ***	38.28
BCFU	Dose	0.003 **	9.12
BCFU	Time × Dose	<0.001 ***	29.91
FCFU	Time	<0.001 ***	43.65
FCFU	Dose	<0.001 ***	20.09
FCFU	Time × Dose	<0.001 ***	21.83

DA, dehydrogenase activity; UA, urease activity; CA, catalase activity; PA, phosphatase activity; BCFU, number of bacteria-forming units; FCFU, number of fungal-forming units; Time, exposure duration; Dose, herbicide dose; Time × Dose, interaction between exposure dose and duration. Asterisks (*) indicate significant differences compared to the reference group (post-hoc Tukey HSD, ***—*p* ≤ 0.001, **—*p* ≤ 0.01).

**Table 3:** Alerts structure for fluroxypyr.

Toxicophore Rules			
Acute Aquatic Toxicity Rule	1	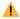	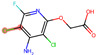
Genotoxic Carcinogenicity Mutagenicity Rule	3	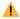	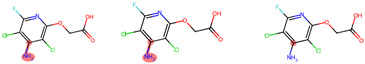
Skin Sensitization Rule	3	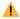	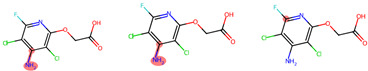
NonBiodegradable	2	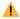	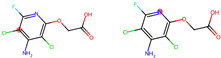
Medicinal Chemistry			
Alarm_NMR Rule	3	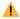	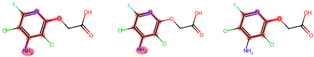

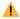
 indicates that the compound triggers the corresponding alert according to the Toxicophore and Medicinal Chemistry Rules.

**Table 4:** Three-dimensional similarity coefficients for fluroxypyr and triclopyr.

Coefficients	Values	Coefficients	Values	Coefficients	Values
TanimotoCombo	1.409	FitTversky	0.902	RefColorTversky	0.788
ShapeTanimoto	0.886	FitColorTversky	0.609	ScaledColor	0.801
ColorTanimoto	0.523	RefTverskyCombo	1.769	ComboScore	1.688
FitTverskyCombo	1.511	RefTversky	0.981	ColorScore	−4.808
				Overlap	583.116

**Table 5:** Docking results.

ZINC ID	2D-Structure	Glide			
		gScore	LE	Evdw	E
ZINC000000156001	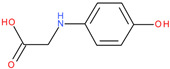	−4.787	−0.173	−19.706	−29.287
ZINC000000085987	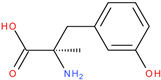	−5.134	−0.367	−18.017	−22.435
ZINC000100289948	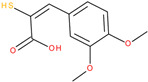	−5.618	−0.351	−21.292	−26.334
ZINC000058855098	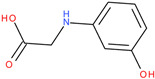	−5.641	−0.246	−20.169	−29.697
ZINC000000150396	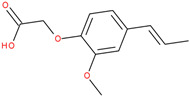	−5.674	−0.355	−22.935	−31.117
ZINC000019518707	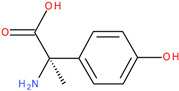	−5.682	−0.437	−21.147	−24.886
Triclopyr	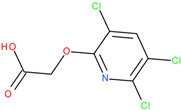	−6.698	−0.478	−25.111	−33.508
Fluroxypyr	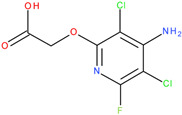	−7.021	−0.468	−21.923	−33.812

**Table 6:** Experimental variants considered in the study.

Experimental	Herbicide Dose Applied
T1	½ DNmax fluroxypyr 125 g L^−1^
T2	DNmin fluroxypyr 160 g L^−1^
T3	DNmax fluroxypyr 250 g L^−1^
T4	2xDNmax fluroxypyr 500 g L^−1^
M	Control (untreated sample)

DNmax—maximum recommended dose; DNmin—minimum recommended dose; 2xDNmax—double the maximum recommended dose; M—control (untreated).

## Data Availability

The data presented in this study are available on request from the corresponding author.

## Supplementary Materials

The following supporting information can be downloaded at: https://www.mdpi.com/article/10.3390/ijms26178209/s1.

## Abbreviations

The following abbreviations are used in this manuscript:

DA	Dehydrogenase Activity
UA	Urease Activity
CA	Catalase Activity
PA	Phosphatase Activity
UTC	Untreated Controls
BCFU	Number of Bacteria-Forming Units
FCFU	Number Of Fungal-Forming Units
ST	Soil Temperature
SC	Soil Electrical Conductivity
SM	Soil Moisture
SOM	Soil Organic Matter Content
SPC	Soil Phosphate Content
