# Supplementary material for: A Comprehensive Environmental and Molecular Strategy for the Evaluation of Fluroxypyr and Nature-Derived Compounds
Source: Int J Mol Sci. 2025 Aug 24;26(17):8209. doi: 10.3390/ijms26178209 (PMC12428653; doi:10.3390/ijms26178209)
Supplement: Supplementary file 1 [file ijms-26-08209-s001.zip › ijms-3815168-supplementary.pdf]

International Journal of *Molecular Sciences*

MDPI

Supplementary Materials

A Comprehensive Environmental and Molecular Strategy for the Evaluation of Fluroxypyr and Nature-Derived Compounds

Ion Valeriu Caraba[1,2], Luminita Crisan[3*], Marioara Nicoleta Caraba[2,4]

[1] Faculty of Bioengineering of Animal Resources, University of Life Sciences "King Mihai I" from Timisoara, Calea Aradului, 119, 300645, Timisoara, Romania, valeriucaraba@usvt.ro. (I.V.C.)
[2] ANAPATMOL Research Center, "Victor Babes" University of Medicine and Pharmacy of Timisoara, E. Murgu, 2, RO 300041, Timisoara, Romania, nicoleta.caraba@umft.ro
[3] "Coriolan Dragulescu" Institute of Chemistry, Mihai Viteazu Blvd., 24, 300223, Timisoara, Romania, lumi_crisan@acad-icht.tm.edu.ro (L.C.)
[4] Cellular and Molecular Biology Department, "Victor Babes" University of Medicine and Pharmacy of Timisoara, E. Murgu 2, Timisoara, 300041, Romania; nicoleta.caraba@umft.ro (M.N.C.)

* Correspondence: L.C. (lumi_crisan@acad-icht.tm.edu.ro)

Table S1. Physicochemical and Medicinal Chemistry parameters; Absorption, Distribution, Metabolism, Excretion, and Toxicity profiles for Fluroxypyr

Physicochemical Property			Medicinal Chemistry		
Molecular Weight	253.970		QED	0.800	☺
Volume	195.380		SAscore	Easy	☺
Density	1.300		GASA	Easy	☺
nHA	5		Fsp^3	0.143	☹
nHD	3		MCE-18	9	☹
nRot	3		NPscore	-0.958	☺
nRing	1		Lipinski Rule	Accepted	☺
MaxRing	6		Pfizer Rule	Accepted	☺
nHet	8		GSK Rule	Accepted	☺
fChar	0		GoldenTriangle	Accepted	☺
nRig	7		PAINS	0	
Flexibility	0.429		Alarm_NMR Rule	3	⚠
Stereo Centers	0		BMS Rule	0	
TPSA	85.440		Chelating Rule	0	
logS	-3.666		Colloidal aggregators	0.042	☺
logP	1.826		FLuc inhibitors	0	☺
logD7.4	1.556		Blue fluorescence	0.124	☺
pka (Acid)	3.511		Green fluorescence	0.101	☺
pka (Base)	1.396		Reactive compounds	0.321	☺
Melting point	234.498		Promiscuous compounds	0.287	☺
Boiling point	309.824				
**Absporption**			**Distribution**		
Caco-2 Permeability	-4.975	☺	PPB	93.50%	☹
MDCK Permeability	-4.566	☹	VDss	-0.747	☹

PAMPA	+++	☹	BBB	---	☺
Pgp inhibitor	---	☺	Fu	2.50%	
Pgp substrate	---	☺	OATP1B1 inhibitor	-	
HIA	---	☺	OATP1B3 inhibitor	++	☹
F20%	---	☺	BCRP inhibitor	---	☹
F30%	---	☺	MRP1 inhibitor	+++	☹
F50%	---	☺	BSEP inhibitor	---	☹
Metabolism			Excretion		
CYP1A2 inhibitor	---		CL_{plasma}	5.125	
CYP1A2 substrate	--		T1/2	1.273	
CYP2C19 inhibitor	---		Toxicophore Rules		
CYP2C19 substrate	---		Acute Aquatic Toxicity Rule	1	⚠
CYP2C9 inhibitor	--		GenotoxicCarcinogenicity Mutagenicity Rule	3	⚠
CYP2C9 substrate	+++		NonGenotoxic Carcinogenicity Rule	0	
CYP2D6 inhibitor	---		Skin Sensitization Rule	3	⚠
CYP2D6 substrate	---		Aquatic Toxicity Rule	0	
CYP3A4 inhibitor	---		NonBiodegradable	2	⚠
CYP3A4 substrate	---		SureChEMBL Rule	0	
CYP2B6 inhibitor	++		FAF-Drugs4 Rule	0	
CYP2B6 substrate	---				
CYP2C8 inhibitor	--				
HLM Stability	---				
Toxicity					
hERG Blockers	0.052	☺	A549 Cytotoxicity	0.011	☺
hERG Blockers (10um)	0.124	☺	Hek293 Cytotoxicity	0.068	☺
DILI	0.982		BCF	1.448	
AMES Toxicity	0.304	😐	IGC50	2.756	
Rat Oral Acute Toxicity	0.442	😐	LC50DM	3.682	
FDAMDD	0.146		LC50FM	2.934	
Skin Sensitization	0.803		Tox21 Pathway		
Carcinogenicity	0.329		NR-AhR	-	
Eye Corrosion	0.033		NR-AR	---	☺
Eye Irritation	0.971		NR-AR-LBD	---	☺
Respiratory	0.696	😐	NR-Aromatase	--	☺
Human Hepatotoxicity	0.609	😐	NR-ER	---	☺
Drug-induced Nephrotoxicity	0.689	😐	NR-ER-LBD	---	☺
Drug-induced Neurotoxicity	0.678	😐	NR-PPAR-gamma	---	☺
Ototoxicity	0.581	😐	SR-ARE	-	
Hematotoxicity	0.520	😐	SR-ATAD5	---	☺
Genotoxicity	0.950		SR-HSE	--	☺
RPMI-8226 Immunitoxicity	0.037	☺	SR-MMP	--	☺
			SR-p53	-	

*The prediction probability values for the classification endpoints are: 0-0.1 (---); 0.1-0.3 (--); 0.3-0.5 (-); 0.5-0.7 (+); 0.7-0.9 (++); 0.9-1.0 (+++). The relationships corresponding to the three labels are as follows: excellent (green); medium (orange); poor (red).

Table S2. 3D similarity coefficient for top selected natural compounds

Name	Rank	TC	ShT	CoT	FTvC	FTv	FCoTv	RTvC	RTv	RCoTv	CoS	O
ZINC000058855098	1	1.479	0.880	0.599	1.836	1.039	0.797	1.558	0.852	0.706	-5.631	546.567
ZINC000001556709	2	1.452	0.882	0.570	1.620	0.894	0.726	1.711	0.985	0.726	-5.822	641.867
ZINC000014818013	3	1.440	0.952	0.487	1.596	1.000	0.596	1.677	0.950	0.728	-5.911	613.621
ZINC000001762244	4	1.434	0.894	0.540	1.749	0.944	0.805	1.565	0.944	0.621	-4.919	611.649
ZINC000084154632	5	1.411	0.827	0.584	1.962	1.031	0.931	1.418	0.807	0.611	-4.807	516.745
ZINC000000156001	6	1.411	0.798	0.614	1.794	0.985	0.810	1.525	0.807	0.717	-5.717	518.137
ZINC000095099070	7	1.403	0.764	0.639	1.600	0.820	0.780	1.697	0.918	0.779	-6.253	598.598
ZINC000040162797	8	1.392	0.888	0.504	1.688	0.919	0.769	1.558	0.964	0.594	-4.711	626.124
ZINC000002527947	9	1.384	0.787	0.597	1.780	0.984	0.796	1.502	0.797	0.705	-5.624	510.957
ZINC000006091356	10	1.384	0.875	0.509	1.675	1.000	0.675	1.549	0.875	0.674	-5.408	562.821
ZINC000000395647	11	1.378	0.842	0.536	1.599	0.936	0.663	1.630	0.894	0.736	-5.945	577.803
ZINC000096295973	12	1.376	0.883	0.492	1.638	0.935	0.703	1.563	0.941	0.622	-4.961	609.853
ZINC000002573574	13	1.374	0.841	0.534	1.835	1.036	0.799	1.434	0.817	0.616	-4.884	523.369
ZINC000197325244	14	1.364	0.902	0.462	1.695	0.970	0.725	1.487	0.928	0.560	-4.433	599.784
ZINC000072220566	15	1.363	0.859	0.504	1.695	0.982	0.713	1.505	0.873	0.632	-5.038	562.458
ZINC000075581582	16	1.361	0.731	0.630	1.690	0.867	0.823	1.553	0.824	0.729	-5.812	532.361
ZINC000001674125	17	1.361	0.887	0.474	1.559	0.875	0.684	1.622	1.016	0.606	-4.833	664.321
ZINC000100289948	18	1.358	0.849	0.509	1.668	0.894	0.774	1.542	0.945	0.597	-4.732	614.110
ZINC000036559124	19	1.357	0.914	0.443	1.542	0.928	0.614	1.597	0.984	0.613	-4.921	639.761
ZINC000012649959	20	1.349	0.853	0.496	1.551	0.921	0.630	1.619	0.920	0.699	-5.646	596.199
ZINC000004045207	21	1.345	0.853	0.493	1.705	0.947	0.758	1.48	0.895	0.585	-4.632	578.499
ZINC000000143613	22	1.345	0.801	0.544	1.756	0.947	0.809	1.463	0.838	0.624	-4.947	539.914
ZINC000000156571	23	1.344	0.891	0.453	1.638	0.974	0.664	1.501	0.913	0.588	-4.686	589.513
ZINC000000518398	24	1.342	0.784	0.558	1.724	0.962	0.762	1.484	0.809	0.675	-5.383	519.841
ZINC000038858699	25	1.339	0.792	0.547	1.727	0.914	0.813	1.481	0.855	0.626	-4.959	552.263
ZINC000000085987	26	1.337	0.809	0.528	1.616	0.924	0.692	1.557	0.866	0.691	-5.540	559.526
ZINC000062720071	27	1.322	0.804	0.518	1.680	0.899	0.782	1.489	0.884	0.605	-4.794	572.629
ZINC000000159041	28	1.319	0.816	0.503	1.707	0.939	0.768	1.455	0.862	0.593	-4.698	556.098
ZINC000001673598	29	1.316	0.878	0.439	1.457	0.903	0.554	1.647	0.969	0.678	-5.511	630.470
ZINC000040162798	30	1.312	0.818	0.495	1.618	0.860	0.759	1.531	0.944	0.587	-4.651	615.074
ZINC000039189300	31	1.307	0.918	0.390	1.672	1.028	0.644	1.392	0.895	0.497	-3.934	576.062
ZINC000019518707	32	1.305	0.732	0.573	1.630	0.900	0.729	1.525	0.796	0.728	-5.843	512.770
ZINC000019735776	33	1.304	0.729	0.575	1.681	0.843	0.838	1.491	0.844	0.647	-5.124	547.193
ZINC000000486307	34	1.302	0.827	0.475	1.807	1.067	0.740	1.357	0.786	0.571	-4.522	502.017
ZINC000000393397	35	1.302	0.871	0.431	1.760	1.000	0.760	1.366	0.867	0.498	-3.923	557.898
ZINC000000150396	36	1.302	0.811	0.491	1.571	0.870	0.701	1.543	0.922	0.621	-4.950	599.569

*TanimotoCombo (TC); ShapeTanimoto (ShT); ColorTanimoto (CoT); FitTverskyCombo (FTvC); FitTversky (FTv); FitColorTversky (FCoTv); RefTverskyCombo (RTvC); RefTversky (RTv); RefColorTversky (RCoTv); ColorScore(CoS); Overlap (O).
